# Quality of Life in Gastroesophageal Reflux Disease Three Months After Laparoscopic Nissen’s Fundoplication

**DOI:** 10.7759/cureus.10674

**Published:** 2020-09-26

**Authors:** Ajay Kumar, Kunal Raja, Sumeet Kumar, Nadim Quasimuddin, Amber Rizwan

**Affiliations:** 1 Internal Medicine, Jinnah Sindh Medical University, Karachi, PAK; 2 Internal Medicine, Shaheed Mohtarma Benazir Bhutto Medical University, Larkana, PAK; 3 Internal Medicine, Chandka Medical College Hospital, Larkana, PAK; 4 Internal Medicine, B.P. Koirala Institute of Health Sciences, Dharan, NPL; 5 Family Medicine, Jinnah Postgraduate Medical Centre, Karachi, PAK

**Keywords:** gerd-hrql

## Abstract

Introduction: Gastroesophageal reflux disease (GERD) affects various elements of life including sleep, daily and social functioning, and physical and emotional activities. This study aims to determine the impact of laparoscopic Nissen’s fundoplication (LNF) on health-related quality of life.

Methods: This prospective study was conducted in a tertiary care hospital, Pakistan, from Jan 2019 to Feb 2020. Forty-seven participants completed the study. All patients completed the Gastroesophageal Reflux Disease Health-Related Quality of Life (GERD-HRQoL) questionnaire both pre-operatively and three months after LNF.

Results: There was significant difference in pre- and post-operative median Health-Related Quality of Life score (p value: 0.0073). There was improvement in items related to heartburn in HRQoL questionnaire, while questions related to swallowing and bloating either showed no change or worsening.

Conclusion: LNF has a significant impact on health-related quality of life. It is important for the physician to consider the impact of GERD in daily life. Management goals for GERD should also include improvement in quality of life of the patient.

## Introduction

Gastroesophageal reflux disease (GERD) is a common major upper gastrointestinal (GI) problem with 10-20% of the population reporting weekly GERD symptoms. The prevalence of symptom-based GERD varies from 5.2-8.5% in Eastern Asia to 6.3-18.3% in Iran. Pakistan reports a significantly higher prevalence of 22.2% and 24.0% based on hospital-based studies [1]. There has been an increase in the incidence of GERD and its complications, including Barrett’s esophagus and adenocarcinoma of the esophagus, throughout the world [2]. Patients with GERD may present with a broad range of symptoms that can extend beyond the common presenting symptoms of heartburn and regurgitation [3].

GERD symptoms and complications impact general health, such as daily and social functioning and physical and emotional activities. It also influences health-related quality of life (HRQoL) due to frequent interruptions during sleep, work, and social activities [3]. GERD symptoms constitute a significant burden for many patients in disrupting physical, social, and emotional well-being. Impaired HRQoL in GERD patients results in features such as disturbed sleep, reduced vitality, generalized body pain, impaired sex life, and anxiety about the underlying cause of the symptoms. Nocturnal symptoms of reflux disease appear to have a particularly marked impact on HRQoL [4].

Medical treatment with proton pump inhibitors is effective in 90% of patients. Patients often require lifelong medication. Despite there are effective maintenance therapies, compliance with long-term therapy is difficult [5-7]. Kellokumpu, in his study, stated that laparoscopic fundoplication is more effective in the management of short- and medium-term GERD than medical therapy [8].

Despite GERD being prevalent in Pakistan, minimal data is available, particularly on the impact on the quality of life with various treatment options. This study aims to compare the impact on the quality of life pre- and post-laparoscopic fundoplication.

## Materials and methods

**Figure 1:**
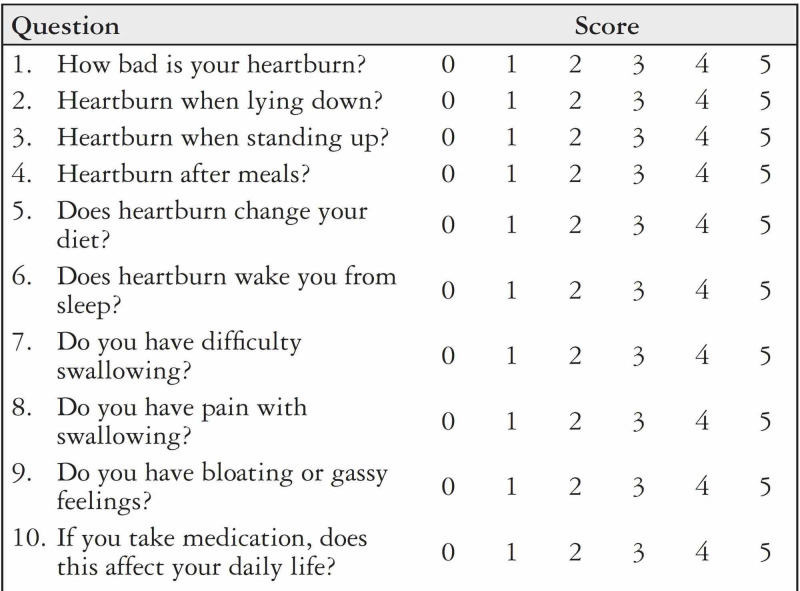
Gastroesophageal Reflux Disorder Health-Related Quality of Life Questionnaire

This prospective study was conducted in a tertiary care hospital, Pakistan, from Jan 2019 to Feb 2020. Fifty patients with GERD were included after informed consent. Patients with a previous history of gastric and esophageal surgery, ulcers, and short esophagus were excluded from the study. All patients underwent thorough clinical evaluation followed by upper endoscopy esophageal manometry and 24-hour pH-metry. All cases underwent laparoscopic Nissen’s fundoplication (LNF) by the same surgeon. After hiatal dissection, hiatal repair was performed. All patients completed the Gastroesophageal Reflux Disorder Health-Related Quality of Life (GERD-HRQoL) questionnaire both pre-operatively and three months after the operation. GERD-HRQoL has ten items with each item that can be scored from 0 (best) to 5 (worst) (Figure 1) [9]. 

Forty-seven participants came for follow-up after three months and three patients were lost to follow up. The numerical value, such as age, was presented as mean and standard deviation. Categorical data were presented, such as gender, was presented as frequencies and percentages. GERD-HRQoL score was presented as median. Median was compared using Mood’s Median test. A P-value of less than 0.05 indicated that there is a difference between pre- and post-operative values of GERD-HRQoL, and the null hypothesis is not valid.

## Results

A total of 47 participants completed the study. The mean age of the participants was 42 ± 11 years. Twenty-three (48.9%) participants were male, and 24 (51.1%) participants were female. Mean stay in hospital after fundoplication was 5 ± 2 days. There was significant improvement in pre- and post-operative median Health-Related Quality of Life score (p value: 0.0073). There was improvement in Items 1-6 and 10. Item 8 and Item 9 worsed after the procedure. There was no change in Item 7 (Table 1).

**Table 1:** Health Related Quality of Life Questionnaire Median Score * means significant result

Health-Related Quality of Life (HRQoL) Questionnaire	Pre-Operative Median score	3 Months Post-Operative Median score	P value
1. How bad is your heartburn?	5 (1-5)	0 (0-2)	0.0073*
2. Heartburn when lying down?	5 (1-5)	0 (0-3)	
3. Heartburn when standing up?	4 (0-5)	0 (0-3)	
4. Heartburn after meals?	4 (0-5)	0 (0-3)	
5. Does heartburn change your diet?	4 (0-5)	0 (0-3)	
6. Does heartburn wake you from sleep?	3 (0-5)	0 (0-3)	
7. Do you have difficulty swallowing?	2 (0-4_	2 (0-4)	
8. Do you have pain with swallowing?	1 (0-3)	2 (0-4)	
9. Do you have gassy or bloating feelings?	1 (0-3)	2 (0-4)	
10. If you take medication, does it affect your daily life?	4 (0-5)	0 (0-3)	

## Discussion

Gastrointestinal esophageal reflux disorder (GERD) is a chronic long-lasting disease, with ability to relapse and develop complications [6]. GERD may cause various symptoms, all of which can lead to disturbance in the patient’s daily life [10]. The negative impact of GERD on quality of life depends on frequency and severity of symptoms. Wikilund et al. showed that even mild symptoms may result in a significant reduction in the wellbeing of a person suffering from GERD [11].

Surgical intervention, such as laparoscopic Nissen’s fundoplication (LNF), is reserved for patients who are intolerant or have relapsed to therapy with medical therapy. It is also offered to non-compliant patients [12]. Salminen, in his study, mentioned that laparoscopic fundoplication provides significant relief in heartburn and regurgitation due to GERD in 84% to 97%. The percentage of patients who are satisfied with the outcome of their laparoscopic fundoplication surgery range from 86% to 96% [13]. 

To the best of our knowledge, this is the first study that compares the health-related quality of life pre- and post-laparoscopic Nissen's fundoplication in a local setting. The study has its limitations as well. The study was conducted in one institute, hence reducing the diversity of sample. The participants were followed up for three months only, because to follow up is fairly common in Pakistan.

In this study, there was a significant improvement in Items 1 to 6 and 10 of the HRQoL questionnaire. Items 1 to 6 relate to symptoms of heartburn. Hamid et al. also showed significant improvement in items related to heartburn in the GERD-HRQoL questionnaire [14]. In this study, there was either no improvement or slight worsening in median score of GERD-HRQoL items related to bloating and swallowing. Difficult in swallowing is common after LNF. Temporary dysphagia occurs in up to 70% of patients after Nissen fundoplication. There may be two possible reasons for dysphagia in patients undergoing LNF: edema at the gastroesophageal junction (GEJ) or transient esophageal hypomotility. In most cases, dysphagia usually resolves within two to three months without any intervention. However, in 3% to 24%, dysphagia persists after LNF [15]. Bloating may also increase after laparoscopic fundoplication. This is mostly due to the increase in basal and nadir pressures at the lower esophageal sphincter, which may increase the symptoms of bloating and fullness. Accidental peri-operation injury to vagal fibres may increase bloating, however it is extremely rare [16]. Anvari et al. observed that patients who had severe bloating pre-fundoplication benefitted the most from the procedure, while patients who had no or minimal bloating preoperative may experience worsening of bloating.

Minimally invasive surgery, such as LNF, have advances the surgical treatment of GERD. Various studies have shown that functional result of both laparoscopic anti-reflux procedures and open surgery are comparable. Laparoscopic procedures have the advantage of significantly lower postoperative morbidity and shorter hospital stay. Minimally invasive surgeries, however, are associated with complications and it is necessary that we manage this complication to ensure maximum long-term benefit of laparoscopic procedures [17].

## Conclusions

There is significant improvement in health-related quality of life three months after LNF. There was significant improvement in the heartburn component of GERD-HRQoL component of the questionnaire, while there was either no change or worsening of median scores related to bloating and swallowing. It is important for physicians to address the impact of GERD on quality of life and daily routine. Management should focus on not only alleviating the symptoms but improving quality of life as well.
